# Supplementary material for: Fake news stance detection using selective features and FakeNET
Source: PLoS One. 2023 Jul 31;18(7):e0287298. doi: 10.1371/journal.pone.0287298 (PMC10389754; doi:10.1371/journal.pone.0287298)
Supplement: Attachment — Submitted filename: PlosOne_FakeNews_Reply_Letter_5_4_2023.pdf [file pone.0287298.s001.pdf]

Manuscript ID: PONE-D-23-02497

Article title: Fake News Stance Detection Using Selective Features and FakeNET

April 24, 2023

Dear Editor,

First of all, we would like to extend our sincere gratitude to Editor, for allowing a re-submission of our manuscript, with an opportunity to address the reviewer's comments. We greatly appreciate the Reviewers' time spent reviewing the paper and assisting in its improvement. These comments are very helpful for revising and improving the paper, as well as the important guiding significance to the other research. We have studied the comments very carefully and made the corrections that we hope to meet with the final approval. We have done our best to modify the paper to reflect all the comments made by anonymous reviewers. These changes are fully addressed in this reply letter.

Below, we provide point-to-point responses explaining how we have addressed each of the Reviewer's comments. For clarity, our responses have been written in blue color.

REVIEWER 1

Concern 1

Introduction section is not well organized. Long paragraphs should be divided into short.

Response: We are thankful for your feedback and appreciate your comments. Respected reviewer, we have revised the Introduction section as per your valuable suggestions.

Concern 2

Comparison with state-of-the-art techniques required.

Response: Thank you very much for the positive feedback. Respected reviewer, We have updated the comparison table with SOTA models. Furthermore, we have added another Table 7 to compare the performance of the proposed FakeNET model with SOTA research work published in 2023.

Performance Comparison with Additional Models For showing the superior performance of the proposed model, this study selected several models reported in [63] and in [81]. The complete comparison in terms of accuracy, f-score, f-agree, f-disagree, f-discuss, and f-unrelated is shown in Table 6. Results show that the proposed ensemble model shows far better results than these models and obtains the highest accuracy and F-score values both average F-score and individual F-score for each class. The comparison of aggregated FNC Score with research published in 2023 is shown in Table 7.

Table 6: Performance comparison of FakeNET model with state-of-the-art approaches.

Model Name	A	F	F-agree	F-disagree	F-discuss	F-unrelated
TalosComb [64]	0.820	0.582	0.539	0.035	0.760	0.994
TalosTree [64]	0.830	0.570	0.520	0.003	0.762	0.994
TalosCNN [64]	0.502	0.308	0.258	0.092	0.0	0.882
Athene [64]	0.820	0.604	0.487	0.151	0.780	0.996
UCLMR [64]	0.817	0.583	0.479	0.114	0.747	0.989
featMLP [64]	0.825	0.607	0.530	0.151	0.766	0.982
stackLSTM [64]	0.821	0.609	0.501	0.180	0.757	0.995
Upperbound [64]	0.859	0.754	0.588	0.667	0.765	0.997
Base [82]	0.871	0.785	0.502	0.272	0.724	0.965
Inf1 [82]	0.869	0.778	0.506	0.278	0.717	0.963
Inf3 [82]	0.870	0.781	0.506	0.288	0.722	0.963
BERT1 [82]	0.875	0.790	0.537	0.314	0.712	0.968
BERT3 [82]	0.877	0.794	0.531	0.340	0.716	0.970
BERT3 + Inf3 [82]	0.879	0.797	0.535	0.312	0.738	0.969
BERT [82]	0.814	0.686	0.431	0.317	0.585	0.916
featMLP [82]	0.871	0.785	0.502	0.272	0.724	0.965
BERTOptimized [82]	0.913	0.861	0.647	0.478	0.800	0.986
XLNet [82]	0.921	0.879	0.686	0.548	0.821	0.986
RoBERTa [82]	0.931	0.891	0.707	0.580	0.845	0.991
**FakeNET**	0.978	0.978	0.860	0.760	0.880	0.990

Table 7: Aggregated FNC-score comparison with research published in 2023.

**Model**	**FNC Score**
GB Classifier [83]	0.795
CNN + GBDT [83]	0.820
CS [83]	0.890
ES + LSTM [83]	0.901
ES + LSTM + AT [83]	0.891
ES + LSTM + AT (optimized) [83]	0.934
**FakeNET**	0.978

Concern 3

Include specific author contributions at the end of the introduction section.

Response: Respected reviewer, thank you very much for your suggestions. PloS one does not allow adding contributions to the manuscript. We have added 'CRediT authorship contribution statement' here for your convenience.

CRediT authorship contribution statement

Xiaoyuan Chen: Funding Acquisition and Validation, **Muhammad Umer:** Conceptualization, Methodology, Software, and Writing – Original Draft Preparation, **Mian Muhammad Ahmed:** Data Curation and Visualization, **Oumaima Saidani:** Investigation and Validation, **Monia Hamdi:** Formal Analysis and Resources, **Khaled Alnowaiser:** Project Administration, **Nihal Abuzinadah:** Supervision, **Imran Ashraf:** Writing – Review & Editing.

Concern 4

Refer the following important works on importance of deep learning models
https://doi.org/10.1016/j.bspc.2022.104549
https://doi.org/10.3390/su15010133

https://doi.org/10.3390/info14020065
https://doi.org/10.1016/j.engappai.2022.105731
Response: Thank you very much for the positive feedback. We have discussed the research works suggested by the worthy reviewer in

Concern 5
In Line 177, The extracted features are fed into the proposed hybrid model which comprises CNN and LSTM" I didn't understand why authors fed selected features to deep learning, because DL has inbuilt feature learning approach?
Response: Respected reviewer, We know that during neuron processing, deep learning models automatically extract features from the overall dataset to get trained on. In this research work, we have added one extra step of significant feature extraction using PCA and chi-square. In this step, we extracted more correlated features from the overall dataset and then let deep learning models decide to extract and use significant features of PCA and chi-square instead of using features from the overall dataset. It can be observed from the results in Table 4 that this extra step of data preprocessing greatly improves the results.

Concern 6
How the data preprocessing unit work? it is not clear.
Response: Respected reviewer, the data-preprocessing unit is based on two parts. The first part consists of data cleaning by tokenization, removing stopwords, lowercase conversion, and stemming. The second step is based on dimension reduction techniques (PCA and chi-square) to extract the significant (more correlated) features among the overall cleaned dataset. Both data preprocessing steps greatly reduce the size of the dataset by extracting only those significant features which are more correlated to target classes. Deep learning models are fed with these significant features for better training and to reduce the complexity of large less correlated features. It can be observed from the results in Table 4 that this extra step of data preprocessing greatly improves the results

Concern 7
Elaborate performance evaluation metrics with equations.
Response: Thank you so much for giving suggestions to improve the quality of the paper. Respected reviewer, we have added the equations of the performance evaluation metrics.

Concern 8
Tuning of the hyperparameters not discussed well.
Response: Thank you so much for giving suggestions to improve the quality of the paper. Respected reviewer, we have added complete layer-wise structure and hyperparameters details of the proposed FakeNET model in Table 3 of the revised manuscript.

Concern 9
Cite the SOTA models in Table 4.
Response: Respected reviewer, We have updated the comparison table with SOTA models. Furthermore, we have added another Table 7 to compare the performance of the proposed FakeNET model with SOTA research work published in 2023.

REVIEWER 2

Concern 1
Authors should edit the manuscript in English.
Response: Respected reviewer, thank you very much for your suggestions. We have proofread the article for English corrections and revised it accordingly.

Concern 2

The Abstract has a lack of flow.
Response: The authors are grateful for your feedback. We have revised the Abstract. The modified Abstract is copied here for your convenience.

The proliferation of fake news has severe effects on society and individuals on multiple fronts. With fast-paced online content generation, has come the challenging problem of fake news content. Consequently, automated systems to make a timely judgment of fake news have become the need of the hour. The performance of such systems heavily relies on feature engineering and requires an appropriate feature set to increase performance and robustness. In this context, this study employs two methods for reducing the number of feature dimensions including Chi-square and principal component analysis (PCA). These methods are employed with a hybrid neural network architecture of convolutional neural network (CNN) and long short-term memory (LSTM) model called FakeNET. The use of PCA and Chi-square aims at utilizing appropriate feature vectors for better performance and lower computational complexity. A multi-class dataset is used comprising 'agree', 'disagree', 'discuss', and 'unrelated' classes obtained from the Fake News Challenges (FNC) website. Further contextual features for identifying bogus news are obtained through PCA and Chi-Square, which are given nonlinear characteristics. The purpose of this study is to locate the article's perspective concerning the headline. The proposed approach yields gains of 0.04 in accuracy and 0.20 in the F1 score, respectively. As per the experimental results, PCA achieves a higher accuracy of 0.978 than both Chi-square and state-of-the-art approaches.

Concern 3

The purpose of the study should come early in the abstract.
Response: Respected reviewer, the Abstract is revised to reflect the objective of the study.

Concern 4

How authors addressed the issues while using PCA including PCA assumption of linear relationships, outliers treatment; normality; interpretation of components, and how to select the optimized number of components.

Response: Respected reviewer, the answer to your these concerns (linear relationships, outliers treatment; normality; interpretation of components) is that we have used robust PCA in this research work rather than linear kernel PCA. Robust PCA is designed to automatically deal with these kinds of problems. While selecting the optimized number of PCA components is done using the 'Scree' plot. We also added these reasons in the paper PCA section. Thank you for your concern to improve the quality of the paper.

Concern 5

Proposed Model: "Filters f with varying kernel size The output is calculated as (c × e), where e is a word embedding, and c is applied to each e.", please clarify this sentence.
Response: Respected reviewer, we have updated the sentence to make it more clear. The updated sentence is: Multiple filters f of different kernel sizes c are applied on each word embedding e and the output is generated as $(c \times e)$". This means that each filter of different kernel sizes is applied to word embedding and generates different text sequences for the FakeNET model to get trained on.

Concern 6

Proposed Model: The FakeNET model comprises a convolutional layer with 64 filters of 5×5 size, why not 3X3 and why not 32 filters?
It is followed by the max pooling layer with a 4-pool size, how the authors have chosen this number?
Each LSTM layer has 100 neurons while the dense layer has 50 neurons, why?
the dropout layer is used with a 0.2 dropout rate, why not 0.4 or 0.5?
The LSTM layer is used with 100 units, why not 50 or 200?

Response: Respected reviewer, we optimized the design regarding these parameters which means that we experimented with more than one architecture and found the current one as the optimal one.

Concern 7

This study used rectified linear unit (ReLU) as the activation, The ReLU activation function has several problems, including vanishing gradients, dead neurons, lack of smoothness, not centered around zero, and output range limitation. How authors addressed these problems?

Response: The authors are grateful for your valuable suggestions. We will address the limitations mentioned by the reviewer and the reasons for choosing the 'Relu' activation function accordingly.

Limitations of Relu that we deal :

ReLU activation function does not suffer from vanishing gradient problems in deep neural networks. This is because the derivative of the ReLU function is constant and does not saturate, unlike the sigmoid and tanh functions, which have small gradients when the input is very large or very small.

In this research, we deal with dead neurons or "dying ReLU" using learning_rate of 0.0001 and batch normalization technique.

As we address earlier that we use learning_rate of 0.0001 to make a slow learning process. This allows the network to learn the optimal slope for each neuron. This slow learning rate adjustment in Relu makes it behave like 'Leaky Relu' and 'PReLU'.

Output range limitation in Relu occurs mostly in regression problems. In classification problems, where we have a discrete set of probabilities, the output range limit problems mostly do not occur.

Reasons for choosing Relu:

Computational efficiency: ReLU activation function is computationally efficient, which means it can be computed quickly, making it ideal for large neural networks with many layers.

Sparse activations: ReLU activation function produces sparse activations, which means that only a subset of the neurons in a layer will be active for a given input. This property can help reduce overfitting and improve the generalization performance of the model.

Nonlinearity: The reLU activation function introduces nonlinearity to the model, which is essential for the model to learn complex, nonlinear relationships between the inputs and outputs.

Sparsity promotes interpretability: Since the ReLU activation function produces sparse activations, it makes the model more interpretable by enabling the identification of the relevant features for a given output.

Overall, the ReLU activation function is a simple yet powerful activation function that provides several benefits during training [**?**].

Concern 8

Line 153-173: LSTM and CNN are used in the present investigation. Authors should use the latest research using LSTM and CNN in various applications including cyber security.

Response: The authors are grateful for your valuable suggestions. We have updated the Introduction section.

Concern 9

The introduction section requires some relevant articles which emphasize the need and use of LSTM and CNN for various types of applications. The authors should add the following works as planetscope nanosatellites image classification using machine learning; cnn based automated weed detection system using uav imagery; smotednn: a novel model for air pollution forecasting and aqi classification; cdlstm: a novel model for climate change forecasting; deep learning based modeling of groundwater storage change; deep learning based supervised image classification using uav images for forest areas classification; insider threat detection based on nlp word embedding and machine learning; dbotpm: a deep neural network-based botnet prediction model; dnnbot: deep neural network-based botnet detection and classification; development of pccnn-based network intrusion detection system for edge computing, to set the narative of the CNN and LSTM utility and wider acceptability in various areas

Response: Respected reviewer, We have added the above-mentioned research works in the related work section of the revised manuscript.

Concern 10

Performance Evaluation Metrics, please write the formulas, and justify if the authors used an F-1 score, F-0.5 or F-2 and why?

Response: The authors are grateful for your valuable suggestions. We have added the formula of each evaluation metric we used in this research. We have used F1 Score in this research because of two reasons. One we need to balance the factualness and comprehensiveness of the model and only F1 Score does that without giving priority to precision or recall. The second reason is that SOTA research techniques make use of the F1 score, in order to judge our proposed model with SOTA models we need to be on the same track.

Concern 11

Add the future scope of the present work.

Response: Respected reviewer, We have added the future work directions at the end of the conclusion and future work section.

In the future, we plan to test the model on large and complex datasets. We also intend to investigate how an ensemble of the machine and deep learning models can improve overall performance.

Concern 12

Discuss the limitations of the proposed research.

Response: Respected reviewer, we have added the limitation of the proposed model at the end of the discussion section.

Despite their usefulness, feature reduction strategies have some limitations as well. To get better results, the features in the dataset should be co-related. Additionally, this study is restricted to the English language only, and employing it in any other language having differences in writing style may produce very different results.

Concern 13

The conclusion should be rephrased with more clarity and major takeaways from the work.

Response: Respected reviewer, We have updated conclusion section.

REVIEWER 3

Concern 1

This study presents FakeNET, a hybrid deep-learning model that ensembles Convolutional Neural Networks (CNN) and long short-term memory (LSTM) with both Principal Component Analysis (PCA) and Chi-square feature reduction approaches. The result shows that FakeNet achieves excellent accuracy, Precision, recall, and F1 score when trained with (PCA) based features. Also, in comparison with other deep learning FakeNet achieves good complexity success. This is good research work and is technically sound. However, there is minor work to be made, such as the update of the references to reflect the 2021-2023 published works on fake news detection.

Response: The authors appreciate your valuable feedback to improve the quality of the manuscript. We have improved the Introduction and Related Work sections by adding recent research works.
